# Smart Steering Wheel Prototype for In-Vehicle Vital Sign Monitoring

**DOI:** 10.3390/s26020477

**Published:** 2026-01-11

**Authors:** Branko Babusiak, Maros Smondrk, Lubomir Trpis, Tomas Gajdosik, Rudolf Madaj, Igor Gajdac

**Affiliations:** 1 Department of Electromagnetic and Biomedical Engineering, Faculty of Electrical Engineering and Information Technology, University of Žilina, 010 26 Žilina, Slovakia; branko.babusiak@uniza.sk (B.B.); lubomir.trpis@feit.uniza.sk (L.T.); 2 Department of Design and Mechanical Elements, Faculty of Mechanical Engineering, University of Žilina, 010 26 Žilina, Slovakia; tomas.gajdosik@uniza.sk (T.G.); rudolf.madaj@uniza.sk (R.M.); igor.gajdac@uniza.sk (I.G.)

**Keywords:** driver drowsiness detection, dry-contact ECG, in-vehicle vital sign monitoring, sensor fusion, smart steering wheel

## Abstract

Drowsy driving and sudden medical emergencies are major contributors to traffic accidents, necessitating continuous, non-intrusive driver monitoring. Since current technologies often struggle to balance accuracy with practicality, this study presents the design, fabrication, and validation of a smart steering wheel prototype. The device integrates dry-contact electrocardiogram (ECG), photoplethysmography (PPG), and inertial sensors to facilitate multimodal physiological monitoring. The system underwent a two-stage evaluation involving a single participant: laboratory validation benchmarking acquired signals against medical-grade equipment, followed by real-world testing in a custom electric research vehicle to assess performance under dynamic conditions. Laboratory results demonstrated that the prototype captured high-quality signals suitable for reliable heart rate variability analysis. Furthermore, on-road evaluation confirmed the system’s operational functionality; despite increased noise from motion artifacts, the ECG signal remained sufficiently robust for continuous R-peak detection. These findings confirm that the multimodal smart steering wheel is a feasible solution for unobtrusive driver monitoring. This integrated platform provides a solid foundation for developing sophisticated machine-learning algorithms to enhance road safety by predicting fatigue and detecting adverse health events.

## 1. Introduction

The integration of health monitoring systems into vehicles has gained increasing attention in recent years [1]. Continuous monitoring of vital signs during driving can provide early warning of potential health issues and help prevent accidents caused by drowsy driving or sudden medical emergencies. The transformation of the car from a mere mode of transport to a platform for continuous, unobtrusive health assessment allows the integration of personalized medicine into daily routines, particularly benefiting an aging population and individuals with chronic medical conditions [2,3].

Driver fatigue and various underlying health conditions collectively represent substantial and frequently underestimated threats to road safety. The consequences of these factors are severe and far-reaching, impacting global public health and safety. In the United States, the National Highway Traffic Safety Administration estimated that drowsy driving accounts for approximately 100,000 crashes annually, leading to 40,000 injuries and 1550 deaths [4]. Research from organizations such as the AAA Foundation for Traffic Safety and the National Sleep Foundation suggests that the actual prevalence is much higher. Some estimates indicate that drowsy driving could be a factor in 10–20% or even more of all crashes, and is significantly higher for fatal crashes (e.g., 16.5% or more of fatal crashes) [5,6]. Drowsy driving incidents exhibit distinct temporal patterns, frequently peaking between midnight and dawn, with a secondary, smaller peak in the afternoon [7]. These periods align with the human circadian rhythm, when the body naturally experiences a decline in vigilance. Such incidents predominantly occur in non-urban areas on high-speed roadways and are frequently characterized as single-vehicle roadway departures or collisions with parked vehicles [8].

Common strategies to combat drowsiness while driving include the consumption of caffeine, opening windows, stretching, and taking rest breaks [9]. However, the effectiveness of these strategies depends on the driver’s awareness of their impaired state and their ability or willingness to implement these measures. Additionally, drivers may inaccurately assess their level of sleepiness by underestimating or overestimating it [10]. Therefore, continuous assessment of the driver’s state in real-time is crucial for enabling prompt interventions, such as alerting the driver or engaging automated driving technologies. Driver drowsiness detection systems are broadly categorized into four types: subjective, behavioral, vehicle-based, and physiological. Subjective measures, which rely on self-report scales [11,12], are impractical for continuous monitoring and often exhibit low correlation with objective indicators [13]. Behavioral approaches utilize cameras to monitor visual cues, such as eye closure and head nodding; however, their accuracy is frequently compromised in real-world settings by variable lighting, occlusions, and image quality [7,14,15,16,17,18]. In contrast, vehicle-based measures non-intrusively analyze driving data such as steering corrections and lane deviation [19,20,21]; however, they lack specificity, as these patterns can be confounded by numerous factors other than drowsiness [22]. The most reliable methods are physiological, directly assessing a driver’s internal state through signals such as electroencephalography (EEG), the gold standard for detecting drowsiness-related brainwave changes [23], as well as electrocardiography (ECG), photoplethysmography (PPG) and electrooculography (EOG). These physiological signals can detect the onset of drowsiness with high accuracy, often preceding overt behavioral signs [24,25,26,27,28,29]. Despite their reliability, the primary challenge of physiological methods remains their intrusiveness, motivating a significant research thrust toward developing unobtrusive, vehicle-integrated alternatives by embedding physiological sensors into vehicle components, such as seats or steering wheels, without requiring a direct attachment to the driver’s body.

Steering-wheel-integrated systems represent a particularly promising and minimally invasive method for detecting driver drowsiness. These systems utilize continuous physical contact between the driver’s hands and the steering wheel to collect a variety of data streams. They can directly measure physiological signals such as ECG or PPG, from which the heart rate (HR) and its variability (HRV) can be derived. Concurrently, the steering wheel functions as a direct interface for monitoring vehicular parameters and driver behavior, including steering wheel angle and subtle micromaneuvers. Several publications have focused on steering-wheel-based monitoring systems, which can be broadly classified into methods that analyze steering dynamics and those that integrate physiological sensors.

Zhang et al. [30] investigated the use of steering wheel angle (SWA) and its statistical properties to detect fatigue. They extracted time-domain features, such as the standard deviation of the SWA, steering wheel reversal rate, and number of zero-crossings from the driving simulator data. The combination of these features using a support vector machine (SVM) classifier could distinguish between alert and drowsy states with an accuracy of 90%. Arefnezhad et al. [31] used an approach involving analyzing the SWA and velocity data, employing an adaptive neuro-fuzzy system for feature selection and an SVM for binary classification. Tested on 20.5 h of driving simulator data, the system achieved 98.12% accuracy, demonstrating the efficacy of advanced machine learning in drowsiness detection. Zhu et al. [32] developed the Multi-Layer Adaptive Driver Fatigue Monitoring model, processing steering wheel signals through adaptive algorithms and feature selection. Validated with a random forest model, it achieved 93.71% accuracy, with the steering wheel angle and lateral acceleration identified as key features.

A notable contribution by Jung et al. [33] proposed a real-time driver health condition monitoring system utilizing an ECG sensor with electrically conductive fabric electrodes located on the steering wheel. The driver’s health conditions, including normal, fatigued, and drowsy states, were analyzed by evaluating the HRV in both the time and frequency domains. The performance of the monitoring system was assessed through a continuous 2 h driving test of two participants. In 2015, Lourenco et al. [34] introduced a system for the continuous acquisition of ECG signals by sewing conductive fabric into a typical steering wheel cover. This prototype, known as CardioWheel, utilizes a pair of conductive fabric electrodes and combines both unsupervised and supervised machine learning algorithms to process ECG data and recognize the driver’s identity. Warnecke et al. [35] explored a sensor fusion approach to achieve robust heartbeat detection in noisy and dynamic driving environments. This study used four types of sensors: ballistocardiography (BCG), ECG, PPG, and image-based PPG. The steering wheel of the driving simulator was equipped with a pair of dry copper ECG electrodes and PPG sensors. Twelve healthy volunteers participated in an 11 min recording session, which included simulated driving. The results consistently showed that fusing multiple sensors significantly enhanced the reliability of heartbeat detection, with the highest score achieved by a specific sensor pair (ECG and BCG) at 93.08%. Subsequently, the same authors [36] replaced the rigid copper ECG electrodes with specially designed flexible and thin ECG electrodes made of polyurethane, which were screen-printed with silver paste to cover the lower half of the steering wheel, thereby enhancing both contact and comfort. The ECG signals from the steering wheel collected from 19 healthy subjects across four scenarios (rest, city driving, highway driving, and rural driving) displayed distinctive signal features compared to the reference ECG. Although these signals were noisier than the reference ECG, the system successfully provided usable heart rate data for a substantial portion of the driving time (approximately 45.62%). Botta et al. [37] proposed a system designed for the real-time monitoring of a driver’s health using ECG and PPG signals. This system features four rigid silver/silver chloride ECG electrodes and two PPG sensors, all strategically placed on the steering wheel. In the laboratory setup, experimental tests conducted during driving simulations with a single subject demonstrated the system’s flexibility and reliability under various simulated driving conditions. Further work by Khan et al. [38] introduced a design for a driver health monitoring system featuring ECG sensor electrodes positioned on both the left and right sides of the upper and lower sections of the steering wheel, where drivers typically grip the wheel most frequently while driving. The front and rear electrodes were used interchangeably for ECG recording, thereby ensuring driver comfort. Moreover, the proposed steering wheel design incorporates PPG and temperature sensors to detect the oxygen saturation, heart rate, and body temperature. In laboratory tests with ten participants, the authors reported a mean absolute error of 1.5 bpm for heart rate measurements from the ECG sensor, 1.3% for oxygen saturation, and 0.58 °F for temperature sensors when compared to standard medical devices.

The first approach focuses on analyzing vehicle motion data, primarily obtained from steering wheel signals (SWS), such as the steering wheel angle and velocity. These systems employ machine learning models and advanced feature selection techniques to identify patterns in the steering behavior that indicate drowsiness. The distinct advantage of this method lies in its robustness and the continuous availability of data, as steering inputs are an intrinsic and constant aspect of driving. However, the core limitation is that SWS data provide only an indirect measure of the driver’s state. Changes in steering behavior can be ambiguous; they may result from driver fatigue but could be caused by external factors such as complex road geometry, traffic conditions, or a response to an unexpected event. This makes it challenging to distinguish between true fatigue-induced performance degradation and a normal driving response. The second approach utilizes physiological sensors embedded in the steering wheel to directly assess a driver’s biological state. Systems employing conductive fabric or printed electrodes have proven to be effective in capturing ECG signals from the driver’s hands. The main advantage of this method is its ability to offer direct insights into the driver’s autonomic nervous system through metrics such as HRV. This enables the direct evaluation of physiological states such as fatigue, stress, and drowsiness, which are precursors to performance decline. In addition, multisensory systems that integrate ECG with PPG and other biosensors have been proposed to create a more comprehensive health profile for the driver. However, this approach has significant limitations in terms of the signal quality and reliability. ECG and PPG signals are highly susceptible to motion artifacts, poor hand contact, varying grip pressure, and skin conditions such as sweat. Studies have quantified this issue, revealing a low signal-to-noise ratio (SNR), indicating that reliable physiological signals may be available for less than 50% of the total driving time, particularly during active steering maneuvers.

An analysis of the current literature reveals a notable research gap, as the two sensing modalities (physiological and steering dynamics) were primarily examined independently. Physiological systems provide direct insights but lack robustness, whereas motion-based systems are robust yet lack direct physiological validation. This separation represents a missed opportunity, as the two approaches possess highly complementary strengths and weaknesses. For instance, a physiological signal such as an ECG is the most reliable during periods of low steering activity (e.g., on a straight highway), precisely when motion-based metrics are the least informative. Conversely, during active steering maneuvers, when ECG signals are the most corrupted, SWS data become rich in information regarding driver performance. Therefore, there is a compelling need for a hybrid monitoring system that integrates physiological and vehicle motion measurements. Such a system can capitalize on its complementary nature to achieve a more accurate, robust, and context-aware assessment of the driver state than is possible using either approach alone. To address these limitations, this study presents an innovative smart steering wheel prototype designed for the unobtrusive monitoring of health and drowsiness. This system builds on previous work [39] by incorporating significant enhancements in the design, sensor technology, and signal processing to achieve superior performance and user experience. The core contribution of this work lies in its multimodal sensing approach, which integrates a dry-contact one-lead electrocardiogram, photoplethysmography, and inertial measurement unit (IMU) directly into the steering wheel.

## 2. Materials and Methods

This section provides a detailed description of the mechanical construction design and overall sensor platform integrated into the steering wheel. This platform uniquely combines sensors that operate independently of a car’s electronics.

### 2.1. Mechanical Construction Design

**Figure 1:**
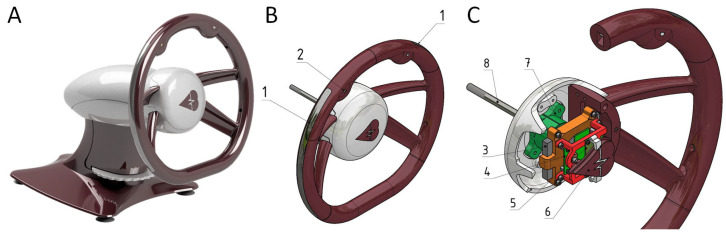
Structural overview of the smart steering wheel prototype. (**A**)—Overall design of the smart steering wheel, (**B**)—Steering wheel rim with embedded sensors, (**C**)—Core section of the steering wheel housing.

A sensory steering wheel was designed and constructed using Autodesk Inventor, a 3D CAD program (Autodesk, San Francisco, CA, USA). Once the design and construction were finalized (Figure 1A), a physical prototype was primarily manufactured using 3D printing. The prototype was mounted on a static base to facilitate the collection of basic datasets in a laboratory setting. The constructed prototype comprises two main components.

The primary component is the steering wheel rim, which contains sensors and electronics (Figure 1B). Embedded within the rim are two electrodes for capturing the electrocardiogram signal (1) and a photoplethysmography sensor (2). The design and positioning of the electrodes ensure they maintain continuous contact with the driver’s hands, even when the grip shifts from the typical “quarter to three” position. The ECG electrodes (1) are located on the lateral sides of the steering wheel (corresponding to the 9 and 3 o’clock positions). They feature an elongated, curved surface fabricated using stainless steel with a matte-polished surface. This specific geometry wraps around the rim profile to maximize the contact area with the driver’s palms, thereby tolerating minor changes in hand placement. The PPG sensor (2) is located on the left side of the rim from the driver’s viewpoint, allowing easy access to the left thumb while ensuring consistent contact between the left hand and ECG electrode. The core of the steering wheel rim (Figure 1C) houses a power battery (3), a main board (4), an LED board (5), and an activation button (6) with a visual indicator. The rim is attached to the steering column housing (11) via a steering hub (7) and secured in place with a bolt (8). Hub (7) is interchangeable, enabling the steering wheel to be installed in various vehicles by adapting the hub design.

**Figure 2:**
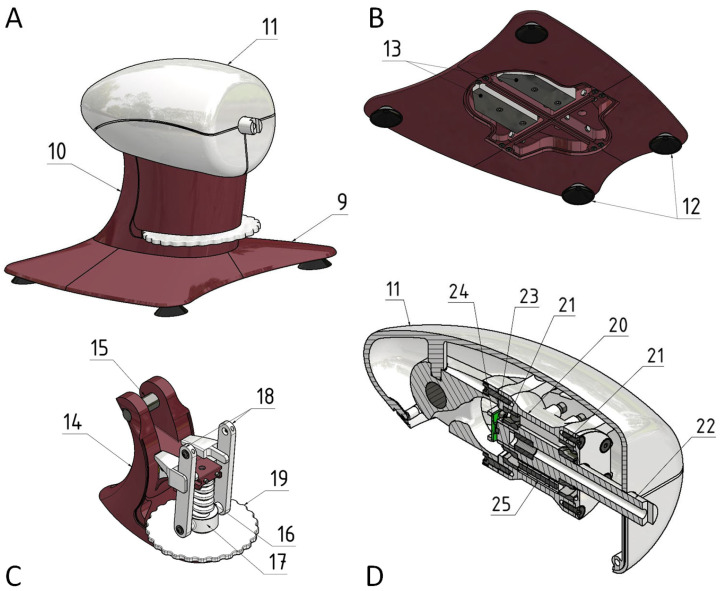
Structural components of the static console of the smart steering wheel system. (**A**)—Perspective view of the steering wheel base, (**B**)—Bottom view of the steering wheel base, (**C**)—Support column of the steering wheel base, (**D**)—Steering housing assembly.

The second principal component is the static console (Figure 2), which consists of a base (9), a support column (10), and a steering housing assembly (11). Base (9) provides a solid foundation for stable placement of the entire assembly on a firm surface (e.g., a table). It is equipped with suction cups (12) to enhance the overall stability and a steel counterweight (13) positioned to shift the center of gravity of the system toward the rear, preventing the steering wheel from tipping forward toward the driver. The support column (14) is mounted on top of the base (9) and secured using screws. Within this column, the steering housing assembly (11) is pivotally attached to pin (15). The height of the steering wheel can be adjusted using a mechanism that includes a trapezoidal-threaded screw (16), nut (17), linkages (18), and an adjustment knob (19). The vertical adjustment range of the steering wheel spans from 0 mm to 135 mm, whereas its angular tilt range extends from 0°to 25°. At the heart of the steering housing (11) lies the main housing body (20), which houses the rolling bearings (21). The steering shaft (22) is mounted on the bearings. At the shaft end, a magnet (23) is affixed, whereas a magnetic sensor (24) is embedded in the housing body (20) to detect the rotational angle of the steering wheel. Additionally, the steering housing features a resistive torque mechanism (25) that generates resistance during the steering wheel rotation. This friction-based mechanism utilizes small rollers that press against the shaft (22) with an adjustable force, thereby simulating a realistic steering resistance.

### 2.2. Electronic Hardware Design

**Figure 3:**
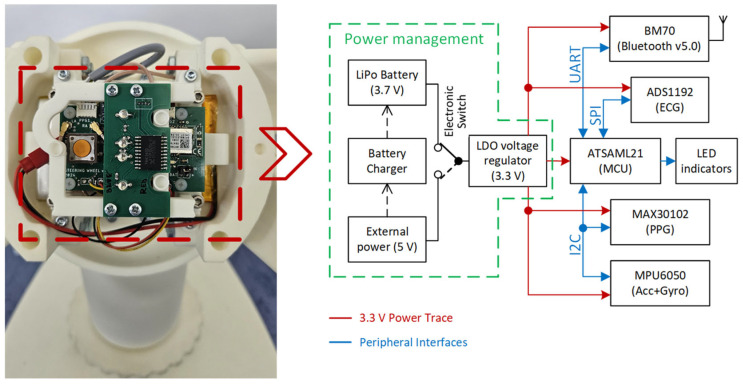
Block diagram of the electronics embedded within the steering wheel hub.

Figure 3 shows a block diagram of the primary electrical components integrated within the steering wheel hub, as shown in Figure 1C. The system architecture was designed with a focus on the energy efficiency and modularity, reflecting the requirements of battery-powered wearable and automotive electronics. The block diagram is divided into three primary sections: power management, communication module, and sensory module. Central to the system is a low-power microcontroller that coordinates the data acquisition, signal preprocessing, and wireless communication.

The central processing unit is the ATSAML21J18B microcontroller (Microchip Technology, Chandler, AZ, USA) [40], a 32-bit ARM^®^ Cortex^®^-M0+ device known for its ultra-low-power consumption and high integration. ATSAML21 was specifically chosen for its suitability in energy-constrained embedded systems. It operates from a single 1.62 V to 3.63 V supply and supports multiple sleep and standby modes with dynamic clock gating to minimize power draw. In its lowest power standby mode, the MCU consumes as little as 0.2 μA. In comparison, active mode consumption remains under 75 μA/MHz, making it ideal for continuous operation from a compact battery source. To accommodate the various peripheral modules integrated into the steering wheel, the MCU offers a rich set of communication interfaces, including SPI, I2C, and UART.

**Figure 4:**
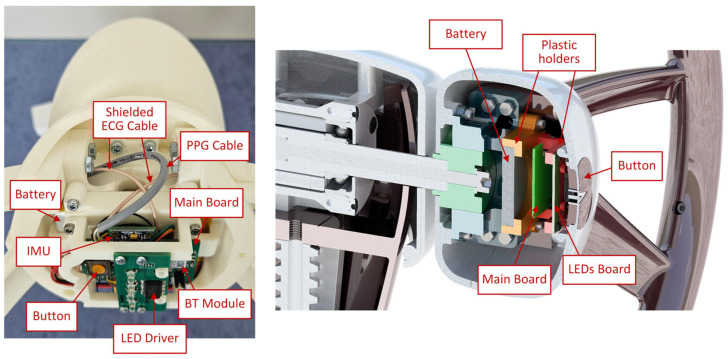
Arrangement of electronic components and cables beneath the front cover of the steering wheel hub.

The power-management subsystem regulates and distributes the voltage levels required by each component. A rechargeable LiPo battery serves as the primary energy source, whereas the battery management circuit handles charging and protection. The system typically operates at 3.3 V, with local regulators providing stable supply levels to sensitive analog components such as the ECG front-end. The sensory module integrates biomedical and inertial sensors that are tightly coupled to the MCU. These sensors provide essential data regarding the driver’s physiological and steering wheel motion states, enabling applications in health monitoring and drowsiness detection. By combining ECG, PPG, and IMU data, the system allows for multimodal analysis, thereby enhancing both robustness and accuracy. The communication module, based on the BM70 BLE chip (Microchip Technology, Chandler, AZ, USA) [41], is optimized for low-power wireless connectivity and supports configurable advertising intervals, secure pairing, and data streaming. This enables the smart steering wheel to interface seamlessly with external devices, such as smartphones, tablets, or in-vehicle systems. The arrangement of electronic components and cables is shown in Figure 4.

The following subsections provide a detailed description of each module, including its functional role, hardware configuration, and integration with the microcontroller unit.

#### 2.2.1. ECG Module

The ECG module is the most crucial part of the smart steering wheel because the electrocardiogram signal serves as the most valuable indicator of cardiovascular health or a reliable sign of driver drowsiness. In the developed prototype, the ECG signal is acquired using two electrodes placed on the lateral sides of the steering wheel. Traditional electrocardiographs typically use at least three electrodes for single-lead ECG recordings. The third electrode, commonly referred to as the driven right-leg (DRL) electrode, fulfills two essential roles. First, it actively suppresses common-mode noise, such as 50/60 Hz power line interference, thereby enhancing signal quality by improving the Common-Mode Rejection Ratio (CMRR) of the amplifier circuit. Second, the DRL electrode provides a return path for bias currents, which is especially important in single-supply systems (e.g., 0 V and +3.3 V), where the input signals must be biased to a mid-supply reference (typically 1.65 V) to ensure the proper operation of the analog front-end. Consequently, achieving high-quality ECG recordings using only two electrodes poses a significant challenge. An additional challenge arises from the use of dry electrodes, which generally exhibit inferior signal quality compared to conventional wet gel-based electrodes.

**Figure 5:**
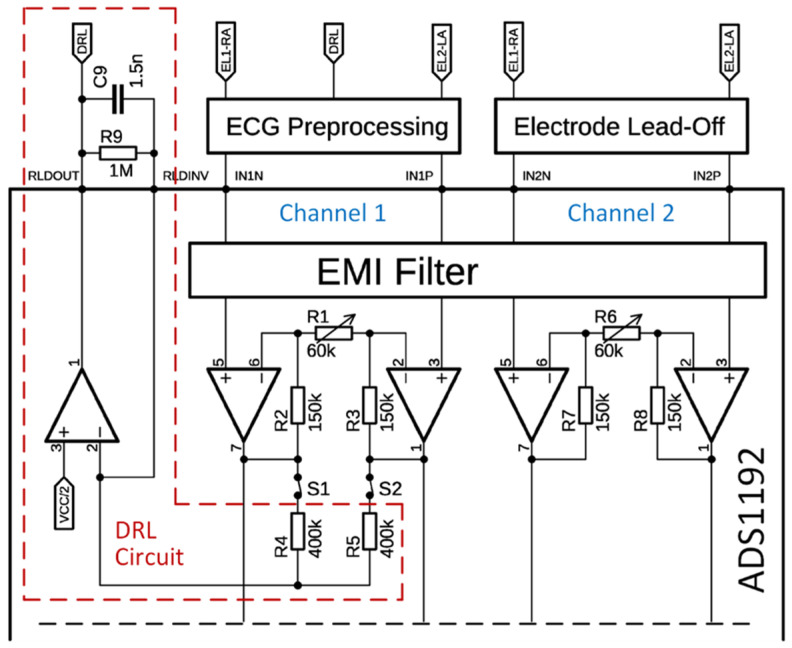
ECG signal-processing block with ADS1192.

The core of the ECG module is the ADS1192 analog front-end (Texas Instruments, Dallas, TX, USA) [42], which supports two analog channels with a 16-bit resolution. The chip integrates a DRL circuit and a programmable gain amplifier (PGA). Channel 1 was used for ECG acquisition, whereas Channel 2 was reserved for electrode lead-off detection (Figure 5). The differential input gain of the PGA was configured using the internal resistors R1 and R6 for Channels 1 and 2, respectively. A resistor value of 60 kΩ corresponds to a gain of 6. The available PGA gain options include 1, 2, 3, 4, 6, 8, and 12. Digitally controlled switches select input signals routed to the DRL circuit. In the configuration shown in Figure 5, switches S1 and S2 connect Channel 1 inputs to the DRL circuit. As Channel 2 does not contribute to the DRL feedback loop, its switches are omitted from the diagram. The gain of the DRL amplifier in the passband is given by the formula [43]:

(1) $$A=\left(-2\cdot \frac{R_{9}}{R_{4}}\right).$$

Using the component values from Figure 5, the resulting DRL gain is *A* = −5. The combination of *R*_9_ and *C*_9_ forms a low-pass filter with a cutoff frequency of approximately 106 Hz.

**Figure 6:**
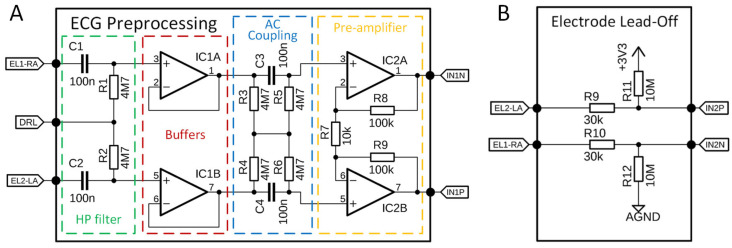
ECG signal processing blocks: (**A**)—lead inputs, AC coupling, and pre-amplifiers; (**B**)—electrode lead-off detection.

In work [39], it is implemented a two-electrode ECG sensing method introduced in [44], which is also adopted in this prototype. In this configuration, the DRL output is injected into the midpoint between two high-value resistors connected to the sensing electrodes (Figure 6A), significantly improving the CMRR.

We used 4.7 MΩ resistors for this purpose. Together with capacitors C1 and C2, these components form a high-pass filter with a cutoff frequency of 0.34 Hz. Despite these measures, the signal remains susceptible to baseline fluctuations because of the dry electrode interface. To further mitigate baseline wandering, an AC coupling circuit is implemented in the preprocessing stage. This circuit is a high-pass filter with the same cutoff frequency (0.34 Hz) and is commonly used in ground-free two-electrode ECG systems [45]. The ADS1192’s built-in PGA can provide up to 12× amplification. An additional preamplifier is used to increase the overall ECG signal gain, which is calculated as:

(2) $$G_{1}=\left(\frac{R_{8}+R_{9}}{R_{7}}+1\right).$$

Using the component values shown in Figure 6A, the resulting gain *G*_1_ is 21. Therefore, the total gain of the ECG signal path becomes:

(3) $$G=G_{1}\cdot PGA$$

This yields a total gain range of 21 to 252, depending on the selected PGA setting.

The lead-off detection feature provides essential information regarding whether both hands are in contact with the electrodes, which is a prerequisite for successful ECG acquisition. This status is monitored in real time, and ECG-based health and drowsiness analysis is suspended when a lead-off condition is detected. ADS1192 supports both the AC and DC methods for electrode lead-off detection. The AC lead-off method utilizes a small excitation signal (6 nA to 22 µA) that falls within the ECG frequency range. Lead-off is detected by measuring the amplitude of this signal in the output spectrum. However, this method requires spectral analysis and is not implemented in the current prototype. Instead, the simpler DC lead-off method is used, which relies on external pull-up and pull-down resistors connected to the positive and negative inputs of Channel 2, respectively (Figure 6B). When an electrode becomes detached, the input saturates, which is detected by on-chip comparators. The comparator outputs are stored in a status register that is read simultaneously with the ECG samples. This allows for real-time detection of lead-off events. The lead-off status was included in the data frame, which is described in a later section. The overall configuration parameters of the ECG module are presented in Table 1.

#### 2.2.2. PPG Module

Compared to the version of the smart steering wheel in work [39], the PPG sensor remains an integral component for capturing cardiovascular signals. The same integrated optical sensor module, MAX30102 (Maxim Integrated, Wilmington, MA, USA) [46], was used due to its compact form factor, high sensitivity, and proven reliability in prior applications. The module is capable of emitting light at two wavelengths (infrared and red) and detecting the corresponding backscattered signal from subdermal blood flow. However, in this iteration, pulse oximetry functionality was deliberately excluded from the system. During experimental validation, it was determined that the signal quality from the red and infrared channels was insufficient to accurately estimate blood oxygen saturation (SpO_2_), particularly under real-world driving conditions where motion artifacts and suboptimal skin contact introduced significant variability. As a result, only the infrared channel was employed for PPG acquisition. IR-based PPG signals continue to provide valuable insights into cardiovascular dynamics, including heart rate and its variability, which remain crucial indicators for assessing driver state and drowsiness. The infrared channel was selected specifically because it provided a more stable and higher-quality signal than the red channel under the measurement conditions. The placement of the sensor within the steering wheel was optimized to ensure consistent and comfortable skin contact.

The MAX30102 sensor was configured via an I2C interface and operated at a clock speed of 400 kHz to enable fast and reliable communication with the microcontroller. The sensor was powered using a 3.3 V supply, consistent with the requirements of other onboard electronics. The LED drive current was set to approximately 20 mA to ensure sufficient tissue penetration while maintaining a low power consumption. For signal acquisition, a 16-bit resolution was achieved by reading two bytes per sample, allowing for an accurate representation of the analog photodetector output. The sampling rate was set at 400 samples per second (SPS), with internal averaging of four consecutive samples applied by the sensor’s built-in processing unit. This resulted in an effective output rate of 100 SPS, offering a suitable balance between temporal resolution and noise reduction for reliable PPG signal analysis under real-time driving conditions. To optimize data retrieval and minimize processor overhead, the sensor’s interrupt output pin was enabled and configured to signal data readiness by pulling the line low, allowing the microcontroller to process new PPG samples promptly upon availability. The overall configuration parameters are listed in Table 2.

#### 2.2.3. IMU Module

To monitor steering dynamics, an MPU6050 inertial measurement unit (TDK InvenSense, San Jose, CA, USA) [47] was integrated into the system. This compact sensor module features a three-axis accelerometer and a three-axis gyroscope, allowing for six degrees of motion sensing. In the current design, only the *z*-axis of the gyroscope was utilized, as it most directly corresponds to the rotational movement of the steering wheel. This axis was found to provide an accurate representation of the steering activity while minimizing unnecessary data overhead from the unused axes. Based on empirical testing, the lowest full-scale programmable range of ±250°/s was chosen for the *z*-axis gyroscope, as the steering wheel rotation speed remains well within this limit during typical driving scenarios. The gyroscopic signal fulfills two essential functions within the driver monitoring system. First, it acts as an indicator for evaluating driver fatigue by monitoring the frequency and consistency of steering microcorrections, which typically decreases as drowsiness sets in. Second, gyroscopic data are instrumental in detecting motion artifacts in ECG signals, especially those caused by steering movements that might disrupt cardiac signal acquisition.

MPU6050 interfaces with the microcontroller using the I2C protocol, operating at a clock speed of 400 kHz. It is powered by a 3.3 V supply, which is consistent with the system’s electrical configuration. The sampling rate was set to 100 samples per second (SPS), which was adequate for capturing both intentional steering actions and subtle microcorrections. To enhance the timing precision and power efficiency, the module’s interrupt pin was activated and configured to alert the microcontroller whenever new data were available, facilitating prompt and synchronized data retrieval. Table 3 lists the overall configuration parameters of the IMU module.

#### 2.2.4. Wireless Communication Module

In the redesigned smart steering wheel, the RN4020 Bluetooth Low Energy (BLE) module in work [39] was upgraded to a more advanced BM70 module (Microchip Technology, Chandler, AZ, USA). This module supports the specifications and features of an integrated chip antenna, enabling a compact design and reliable wireless communication. According to the manufacturer’s datasheet, the BM70 module typically offers a communication range of approximately 50 m under open-air conditions when paired with a smartphone. It is optimized for low power consumption, making it ideal for continuous operation of battery-powered systems. In our design, it operates at a supply voltage of 3.3 V and interfaces with the microcontroller unit (MCU) via a Universal Asynchronous Receiver-Transmitter (UART) interface. The theoretical data throughput of Bluetooth 4.2 is approximately 1 Mbps, which is adequate for transmitting the physiological and motion data collected by the steering wheel’s sensors. Table 4 presents the comprehensive configuration parameters of the Bluetooth module.

#### 2.2.5. Steering Wheel HID Support

In contrast to the version of the smart steering wheel published in work [39], the enhanced prototype incorporates functionality that allows it to operate as a Human Interface Device (HID) compatible with personal computers. This enhancement enables the steering wheel to be used as a game controller for driving simulation games such as the Need for Speed or Euro Truck Simulator.

**Figure 7:**
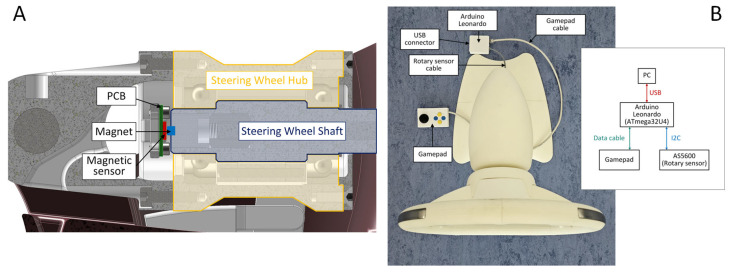
(**A**)—Positioning of the AS5600 magnetic sensor for the detection of steering wheel orientation, (**B**)—HID implementation of smart steering wheel.

To measure the steering angle precisely, an AS5600 (AMS-OSRAM AG, Premstätten, Austria) [48] contactless magnetic rotary position sensor was employed. This sensor was engineered to detect the absolute angular position using a diametrically magnetized magnet affixed to the end of the rotating shaft of the steering wheel (Figure 7A). The AS5600 is mounted on the stationary frame of the steering wheel, maintaining an air gap of approximately 1 mm between the sensor surface and rotating magnet. It offers a 12-bit resolution over a complete 360° rotation, and connects to the processing unit via the I2C communication protocol.

The processing and HID functionality are controlled by an Arduino Leonardo development board, which utilizes an ATmega32U4 microcontroller (Microchip Technology, Chandler, AZ, USA). This microcontroller offers native USB support, enabling it to emulate the USB HID-compliant joystick. The firmware on ATmega32U4 reads the angular position data from the AS5600 sensor and converts it into HID-compatible steering input signals that the game recognizes. Furthermore, the prototype allows for the connection of a simple gamepad extension, which includes a thumb joystick and four push buttons (labeled A, B, X, and Y). These buttons can be assigned to auxiliary game functions such as handbrake, camera control (e.g., look behind), or menu navigation, thereby enhancing the interactivity and realism of the gaming experience. Figure 7B shows the block diagram of the steering wheel used as the HID. This integration of HID functionality demonstrates the flexibility of the smart steering wheel system and its potential for both health monitoring and human–machine interaction in gaming and simulation contexts.

### 2.3. Data Acquisition

Integrated sensors collect data from a smart steering wheel, each of which functions at a specific sampling rate. The ECG signal is sampled at 250 SPS, PPG signal at 100 SPS, and the gyroscope *Z*-axis at 100 SPS. These acquisition parameters were selected to align with standard guidelines for ambulatory physiological monitoring, ensuring sufficient temporal resolution for accurate heart rate variability (HRV) analysis and motion tracking [49,50,51]. Each sensor is linked to a dedicated interrupt pin on the microcontroller, which is activated when new data becomes available. Upon activation, the relevant data are promptly sent to an external device via a Bluetooth connection. In addition to the sensor data, the transmitted packet also includes metadata indicating the contact status between the user’s hands and ECG electrodes, as well as the battery voltage level. The contact status is sent alongside the ECG sample and is crucial for assessing the reliability of ECG signal acquisition. The battery voltage is measured using the microcontroller’s internal analog-to-digital converter (ADC) and transmitted every ten seconds. The data are organized into fixed-length frames, with each data element occupying exactly 4 bytes. This consolidation of distinct sensor outputs into a single, time-correlated data structure constitutes low-level sensor fusion. By ensuring strict temporal alignment between physiological measurements and inertial data, the system allows for the direct correlation of steering events with signal artifacts during post-processing. The format and interpretation of each data element are listed in Table 5.

**Figure 8:**
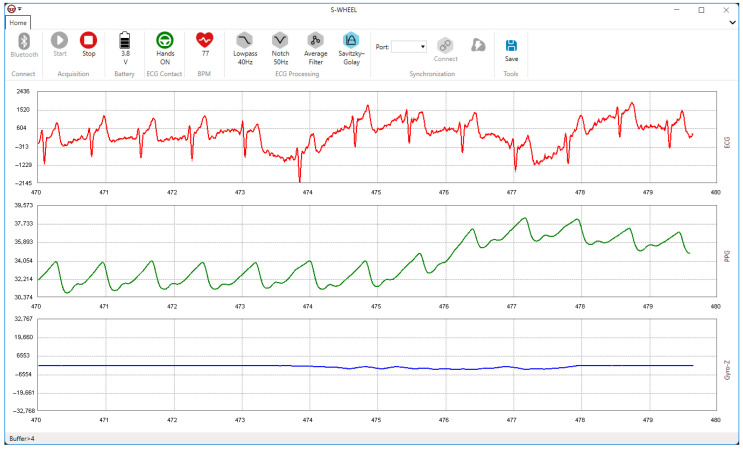
S-WHEEL application window for real-time data visualization and processing. The interface features a control toolbar at the top and three real-time signal plotting panels: the upper panel displays the electrocardiogram (ECG) signal (red trace), the middle panel shows the photoplethysmogram (PPG) waveform (green trace), and the lower panel presents the *Z*-axis gyroscope data (blue trace) representing steering wheel rotation.

A dedicated application for real-time data collection, processing, and visualization was developed using the C# programming language for the Windows operating system (Visual Studio 2026, Microsoft Corporation, Redmond, WA, USA). Although this study primarily focused on the hardware design and functionality of the smart steering wheel, a brief overview of the application is provided here to outline its role in the overall system. The application was successfully tested on various Windows-based platforms, including desktop PCs, laptops, and tablets. Notably, the Microsoft Surface Go 4, equipped with 8 GB of RAM and an Intel N200 processor, serves as the primary testing device in both laboratory settings and real-life driving scenarios. The main application window is illustrated in Figure 8.

**Figure 9:**
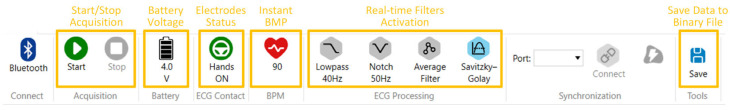
S-WHEEL application toolbar (ribbon) with indicators and buttons.

The software offers real-time visualization and processing of the acquired physiological and motion signals. Its modular architecture is designed to accommodate the integration of future algorithms, such as fatigue detection and health assessment modules. Key indicators, including the ECG electrode contact status, battery level, and instantaneous heart rate (BPM), are displayed in the toolbar section of the application interface, as illustrated in Figure 9. The user interface also facilitates ECG signal enhancement through various filtering options available in the toolbar. All measured data is continuously stored in a temporary binary file. After a measurement session, the data can be permanently saved under a user-defined filename by clicking the “Save” button. It is important to note that only raw (unfiltered) data are saved, ensuring compatibility with post-processing workflows. The format of the saved data follows the structure described in Table 5.

## 3. Results

**Figure 10:**
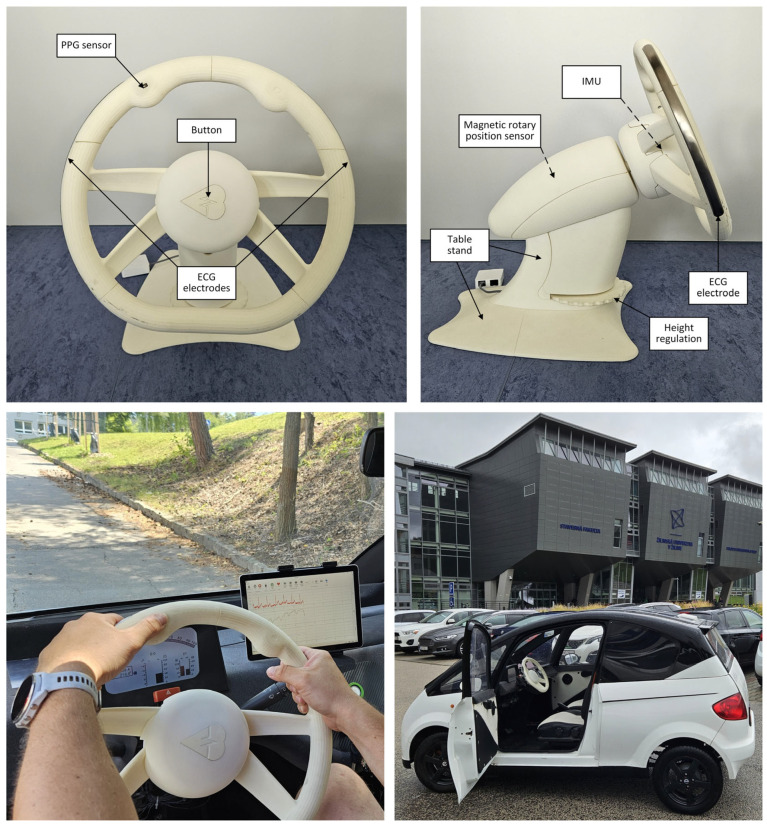
Views of the smart steering wheel prototype on its test stand, indicating the location of the embedded sensors (**Top**). The prototype installed in the Edison electric vehicle (**Bottom**).

A smart steering wheel prototype was fabricated from polyamide 12 (PA12) using selective laser sintering (EOS Formiga P100, EOS GmbH—Electro Optical Systems, Krailing, Germany). This additive manufacturing technique, which builds objects by fusing a polymer powder layer-by-layer with a laser, was selected for its ability to produce parts with high-detail precision. PA12 was chosen because of its excellent mechanical properties and favorable electrical insulating characteristics (Figure 10).

The smart steering wheel prototype was evaluated using both laboratory and real-world tests. This study was conducted in accordance with the Declaration of Helsinki and approved by the Institutional Review Board of the University of Žilina. The evaluation and testing involved a single participant, who provided written informed consent prior to participation. This section presents representative measurements obtained from all the integrated sensors.

In the laboratory setting, signals acquired from the integrated sensors of the prototype were benchmarked against data obtained from certified medical-grade equipment (g.tec Medical Engineering GmbH, Schiedlberg, Austria). The signals from the smart steering wheel prototype were recorded using a tablet running S-WHEEL application. Signal processing and analysis were performed using MATLAB R2023a (MathWorks, Natick, MA, USA). Particular emphasis was placed on the ECG and PPG signals, as they are critical for both assessing cardiovascular health and detecting drowsiness. This study did not repeat the laboratory validation of the IMU, as the sensor configuration and usage remained consistent with those detailed in previous work [39]. For real-world evaluation, the prototype was installed in the Edison electric vehicle [52,53], which was designed and constructed as a custom-built research platform at the University of Žilina. Testing under real-life driving conditions was aimed at verifying the overall functionality of the system and evaluating the performance of the integrated sensors under dynamic and operational scenarios.

### 3.1. ECG Signal Quality Assessment

To assess the quality of the ECG signal, a medical-grade biosignal amplifier, g.HIamp (g.tec Medical Engineering GmbH, Schiedlberg, Austria), served as a reference device. Standard gel-based electrodes were placed on the subject’s wrists to capture Lead I of the 12-lead ECG configuration, with the reference electrode positioned on the right leg. The amplifier was set to a sampling rate of 256 SPS, with a 50 Hz notch filter and a bandwidth of 0.1–100 Hz. In contrast, the smart steering wheel employs two dry electrodes located laterally on the wheel surface, sampling the ECG at 250 SPS, without a third electrode or notch filter.

**Figure 11:**
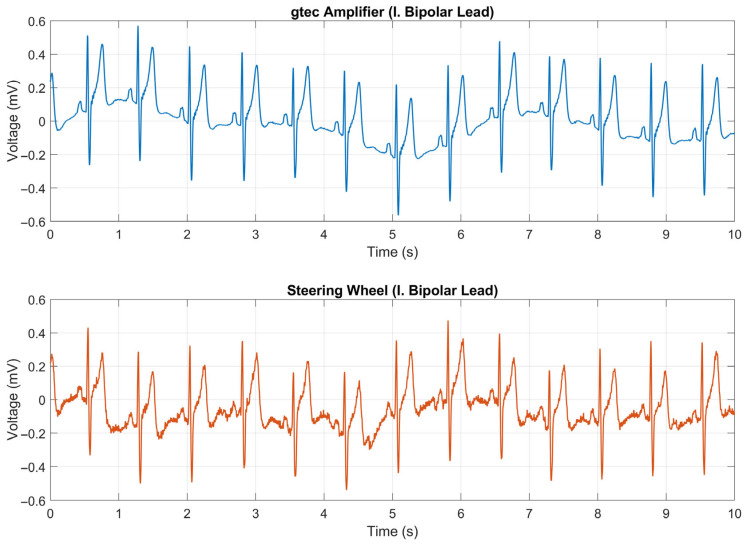
The reference ECG signal captured by g.HIamp (**top**) and the ECG signal obtained from the steering wheel prototype (**bottom**).

Figure 11 displays a 10 s segment of raw ECG signals recorded by both devices. As anticipated, the signal from the steering wheel exhibited slightly higher noise levels due to the absence of the DRL electrode and the use of dry contacts. Nonetheless, key ECG waveform components, including the QRS complex, P wave, and T wave, were clearly discernible.

**Figure 12:**
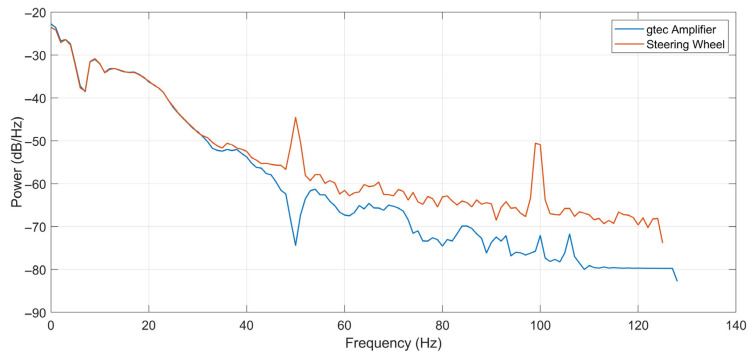
Power spectral density of ECG signals captured by the g.HIamp (blue) and the smart steering wheel (red).

To further examine the noise characteristics, the power spectral densities (PSDs) of both the signals were calculated. As illustrated in Figure 12, the spectrum of the steering-wheel ECG exhibits distinct peaks at 50 Hz and 100 Hz, which are attributed to the power line interference and its harmonics. In addition to these artifacts, the spectral profiles of both signals were nearly identical up to 40 Hz.

Precise identification of R peaks in ECG is a prerequisite for accurate instantaneous heart rate estimation, extrasystole detection, heart rate variability analysis, and drowsiness detection [54]. Accordingly, signal quality was assessed through quantitative evaluation of R-peak detection accuracy in ECG. For a direct waveform comparison, both ECG signals underwent low-pass filtering with a cutoff frequency of 45 Hz. R-peak detection was performed using the BioSPPy toolbox for physiological signal processing in Python 3.11 (Python Software Foundation, Beaverton, OR, USA) [55], which provides several established R-peak detection algorithms. In this study, the Hamilton segmentation algorithm [56], an extension of the widely used Pan–Tompkins method, was selected. The algorithm was applied independently to both the reference ECG (g.HIamp) and the steering-wheel ECG, each with a duration of 1 min.

**Figure 13:**
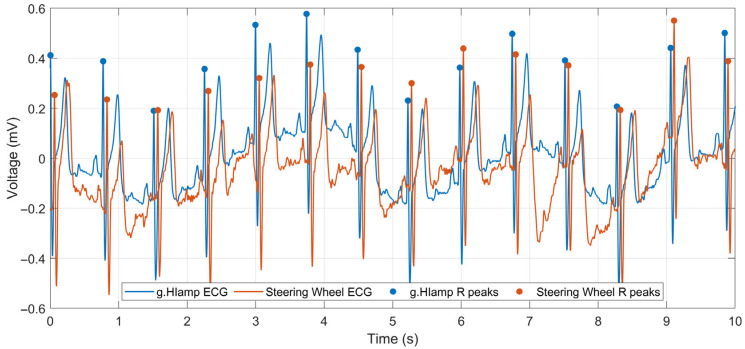
Comparison of ECG signals following low-pass filtering: g.HIamp (blue) and steering wheel (red) with detected R-peaks.

Figure 13 presents a representative first 10 s segment of both signals with the detected R peaks marked. For improved visual clarity, the signals are shown with a constant temporal offset of approximately 55 ms. This synchronization offset was compensated prior to the quantitative evaluation of R-peak detection accuracy. R-peak positions detected in the steering-wheel ECG were compared with the reference R-peak positions using the compare_segmentation function provided by the BioSPPy toolbox. An R peak detected in the steering-wheel ECG was considered a true positive if its position fell within a ±50 ms tolerance window around the corresponding reference R peak; otherwise, it was classified as a false positive. A total of 81 R-peaks were detected in both ECG recordings. The performance metrics of the R-peak detection are later summarized in Table 6. The results presented in Table 6 demonstrate a high precision of peak detection in the steering-wheel ECG when compared with the reference signal, indicating a high signal quality achieved by the proposed measurement system. Although baseline wandering was slightly more pronounced in the steering wheel ECG signal (Figure 13), it remained within acceptable limits, particularly given the use of only two dry electrodes without a ground reference.

### 3.2. PPG Signal Quality Assessment

The quality of the PPG signal acquired by the steering wheel is also assessed. For comparison, a medical-grade g.SpO2 sensor (g.tec Medical Engineering GmbH, Schiedlberg, Austria) was attached to the subject’s right index finger and connected to the g.HIamp system. Simultaneously, the subject’s left thumb was positioned on the PPG sensor embedded in the steering wheel (Figure 10). To mitigate variability in the signal range, both signals were normalized to the [0, 1] interval. Initially, a minor synchronization issue was detected, which was traced back to the actual sampling frequency of the MAX30102 sensor nominally set at 100 SPS. Oscilloscope measurements of the interrupt pin revealed an actual sampling rate of approximately 99.4 SPS, likely due to internal clock inaccuracies.

**Figure 14:**
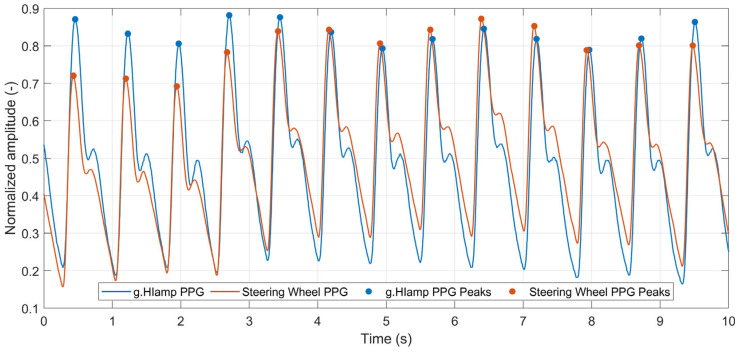
Comparison of PPG signals from the g.SpO2 sensor (blue) and the steering wheel prototype (red) with detected systolic peaks.

Similarly, precise identification of systolic peaks in the PPG signal is fundamental for accurate pulse rate estimation, variability analysis, and drowsiness detection [57]. Consequently, PPG signal quality was assessed through quantitative evaluation of systolic-peak detection accuracy. The systolic peaks were detected using the BioSPPy toolbox for Python 2.2.4. Despite being acquired from different hands and fingers, the signals exhibited high morphological similarity, with closely matching pulse peaks (Figure 14). The figure shows just first 10 s segment of 1 min interval used for quality assessment. The accuracy of systolic peak detection in PPG was evaluated in the same way as for R peaks in ECG. The same 1 min interval was used, so the results are comparable with R peaks results. The number of detected systolic peaks in both PPGs is 80, in contrast to 81 peaks found in the ECG signal. It is caused by a delay of the PPG signal with respect to the ECG signal, so the last PPG peak is outside the 1 min interval. The parameters that reflect performance metrics of the systolic peak detection are summarized in Table 7.

The results presented in Table 7 demonstrate a high precision of peak detection in the steering-wheel PPG when compared with the reference signal, indicating a high signal quality achieved by the proposed measurement system. Consequently, the steering wheel’s PPG sensor provides a signal of sufficient quality for heart rate monitoring, HRV estimation (via interbeat intervals), redundancy in the case of a degraded ECG signal, and basic vascular assessment (e.g., pulse shape and amplitude). These findings indicate that the steering-wheel ECG and PPG provide sufficient accuracy for heart rate estimation, detection of extrasystoles, heart rate variability analysis, drowsiness detection, and basic cardiovascular assessment.

### 3.3. Testing in Real-Life Driving Scenarios

**Figure 15:**
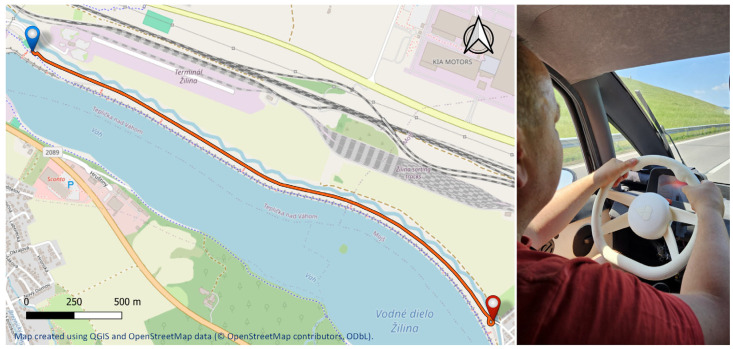
Map of the test route with marked start (blue) and end (red) points used for real-world evaluation of the smart steering wheel prototype.

Real-world testing of the smart steering wheel prototype was conducted on a countryside road located in the vicinity of Žilina, Slovakia (Figure 15). This road segment was selected because of its suitability for controlled testing, offering a combination of light traffic conditions, minimal road-surface irregularities, and a posted speed limit of 90 km/h. The route is approximately 3 km in length and features a relatively smooth asphalt surface, free of significant bumps or sharp turns, thereby allowing consistent signal acquisition from onboard physiological sensors without excessive motion artifacts.

The road features designated parking areas at both the starting and ending points, facilitating a two-way testing scenario in which the vehicle completes a full out-and-back ride with a turnaround at the far end of the route. Each one-way drive along the test segment took approximately 3 min at an average speed of approximately 70 km/h, depending on the traffic conditions. This real-world scenario was designed to validate the functionality of the smart steering wheel and assess the performance of the integrated sensor system under dynamic driving conditions such as vehicle acceleration, braking, steering maneuvers, and road-induced vibrations.

**Figure 16:**
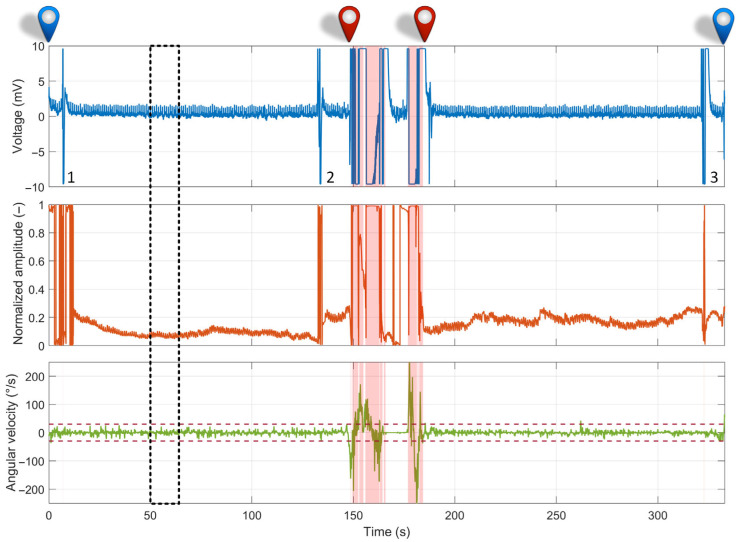
Representative ECG, PPG, and gyroscope signals recorded during real-world driving with the smart steering wheel prototype. The start (blue) and end (red) points of the route are marked; pink highlights indicate electrode lead-off; red dashed line indicates the angular velocity thresholds for steering wheel microcorrections; numbered annotations correspond to driver gestures—turn signals.

A representative measurement of the overall driving route is shown in Figure 16. Signal segments highlighted in pink indicate a loss of contact between the driver’s hands and the electrodes. These occurrences were automatically detected using the lead-off detection feature, as described in Section 2.2.1. Contact loss occurred primarily during the middle portion of the recording, which corresponded to the vehicle’s turnaround phase, during which the driver briefly lost contact with the electrodes multiple times.

The ECG signal contained several signal artifacts, each annotated by a number in the recording (Figure 16). The first artifact (no. 1), located at the beginning of the recording, resulted from a brief loss of contact between one of the electrodes and the driver’s hand, while signaling a turn upon exiting the parking area. The subsequent artifacts have similar origins: artifact no. 2 corresponds to the turn signal used before making the turnaround, and artifact no. 3 corresponds to the signal when returning to the parking area. The PPG signal exhibited a behavior similar to that of the ECG signal, with signal loss occurring during brief movements of the left-hand finger away from the sensor. The gyroscope signal, specifically the *Z*-axis, did not show large steering angle changes during the driving, which was conducted primarily on a straight road segment. The data reflect minor steering corrections within ±30°/s, as indicated in the graph by the two horizontal dashed lines. The largest deviations were observed during the vehicle turnaround, occurring roughly at the midpoint of the recording.

**Figure 17:**
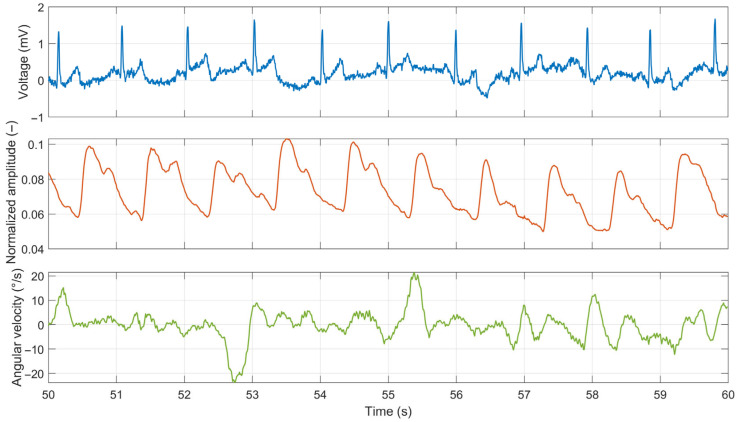
Ten-second raw segment of ECG, PPG, and gyroscope signals recorded during real-world driving with the smart steering wheel prototype.

To illustrate the signal quality, a 10 s raw data segment while driving the test route (dashed black rectangle in Figure 16) is shown in Figure 17.

Digital filtering or preprocessing techniques were not applied during data acquisition. This approach was chosen to evaluate the raw performance of the analog front-end and the mechanical stability of the electrode contact without the masking effects of software enhancement. While this results in signals that exhibit lower quality compared to recordings obtained under laboratory conditions (Figure 13 and Figure 14), the raw data confirms the hardware’s intrinsic robustness. Nevertheless, the ECG signal remained sufficient for reliable R-peak detection. PPG signal quality depends heavily on the placement of the thumb on the sensor. Even slight changes in position can result in signal loss or waveform distortion, making heart-rate estimation unreliable in this setup. Additionally, vibrations during driving may induce micro-movements of the hands on the ECG electrodes and the thumb on the PPG sensor, introducing motion artifacts. This issue is more pronounced in the Edison prototype vehicle, where the development prioritized functionality over ride comfort, including limited suspension and noise isolation.

## 4. Discussion

This study successfully demonstrated the feasibility of a novel smart steering wheel prototype for the unobtrusive, multimodal monitoring of a driver’s vital signs. Our primary findings indicate that integrated sensors can acquire physiological data of sufficient quality for meaningful analysis, laying the groundwork for advanced in-vehicle health and drowsiness detection systems.

The key finding of our laboratory validation is that, despite its inherent design simplifications, the two-electrode dry-contact ECG system captures a signal with high clinical utility. The raw ECG signal exhibited more baseline wander and powerline interference than the medical-grade reference device (Figure 11 and Figure 12). This was an expected outcome due to the absence of a DRL electrode and the nature of the dry electrodes. However, post-processing revealed remarkable preservation of the essential ECG waveform morphology (Figure 13). The precise alignment of R-peaks after filtering confirms the system’s suitability for robust HR and HRV analysis. While actual fatigue classification was not part of this study, these metrics represent critical inputs for the future development of systems capable of assessing the cardiovascular state and drowsiness. Furthermore, successful integration of the PPG sensor provides a secondary data stream. The high morphological correlation between the PPG signal of the prototype and that of a reference sensor validates its use for HR monitoring and as a crucial redundancy measure (Figure 14), particularly when the ECG quality may be compromised by holding the steering wheel with only the left hand. The successful fusion of these physiological sensors with an IMU within a single cohesive prototype represents a significant step toward creating a hybrid monitoring system.

Furthermore, our initial real-world evaluation in the Edison electric vehicle confirmed the prototype’s functionality under dynamic driving conditions. As shown in Figure 16, the system effectively operated on a real road, and the lead-off detection feature successfully identified the periods of hand-off contact during steering maneuvers. The data confirmed that real-world driving introduces significant motion artifacts, particularly from road vibrations and discrete driver actions such as the use of turn signals. These factors degraded the signal quality compared to the laboratory setting (Figure 17). The PPG signal proved highly sensitive to minor thumb movements and pressure changes, occasionally rendering the HR estimation unreliable. Critically, however, the ECG signal, while noisier, remained sufficiently robust for consistent R-peak detection throughout the drive, demonstrating its potential as the primary modality for HRV analysis even in a challenging environment. This robustness is particularly notable given that it was achieved solely through the analog front-end design (differential amplification and analog high-pass filtering) without the application of digital noise suppression or artifact removal algorithms.

Despite these promising results, several limitations of this study remain to be acknowledged. First, the evaluation presented in this study was confined to a single participant to demonstrate the feasibility of the multimodal sensing prototype. The prototype’s performance in a real-world dynamic driving environment, where motion artifacts from steering, road vibrations, and driver repositioning present the most significant challenges, remains to be quantified. The actual robustness of sensor fusion can only be determined through extensive on-road testing involving a diverse cohort (e.g., *N* > 10) with varying ages, genders, and skin characteristics to validate the prototype’s robustness. Second, the reliance on a single test subject limits the generalizability of our findings. We anticipate that physiological diversity will impact signal reliability; for instance, variations in skin hydration levels can significantly alter contact impedance, potentially increasing baseline wander in the ECG signal, while differences in peripheral perfusion and skin tone may affect the amplitude and signal-to-noise ratio of the PPG sensor. Similarly, varying driving behaviors, such as frequent one-handed driving or aggressive steering inputs, are expected to introduce intermittent signal loss and muscle motion artifacts. Factors such as skin condition, hand size, grip pressure, and underlying physiological differences can significantly affect the quality of ECG or PPG signals, necessitating evaluation across a diverse participant pool. A third limitation stems from the hardware design itself; the two-electrode ECG configuration, while minimally intrusive, inherently offers lower common-mode rejection than traditional three-electrode systems, making it more susceptible to environmental noise.

The primary strength of this study is the successful integration of a multimodal sensor array into a functional, non-intrusive prototype. By combining ECG, PPG, and IMU data streams, our system was designed to overcome the weaknesses of single-modality approaches, where physiological systems lack robustness and vehicle-based systems lack specificity. In this sensor fusion framework, the IMU data plays a critical role in artifact management. High-amplitude steering maneuvers detected by the gyroscope (e.g., sharp turns) directly correlate with muscle artifacts and potential electrode instability. By using the IMU signal to flag these high-motion epochs, the system can dynamically exclude or down-weight the corresponding physiological data segments, thereby preventing false alarms in heart rate or drowsiness detection. Furthermore, the IMU contributes independent behavioral metrics (such as steering reversal rates) that, when fused with physiological HRV data, enable a more comprehensive assessment of driver fatigue. Another significant strength is the prototype’s dual-purpose design, which includes HID support. This feature uniquely positions the steering wheel as a valuable tool for conducting controlled, repeatable, and engaging driver fatigue studies using widely available driving simulators. This functionality is particularly valuable for creating large-scale, controlled datasets by recording sleep-deprived subjects during monotonous driving simulations. Such data, which can include metrics such as reaction time, are essential for developing and validating robust machine learning models that fuse physiological signals with steering behavior to not only detect drowsiness but also predict the onset of fatigue. Our findings are in agreement with existing research [33,36,38], which confirms the viability of steering-wheel-based ECG while advancing the field by presenting a more comprehensive, multi-sensor platform.

Our study highlights several clear directions for future research. The next step is to conduct extensive on-road validation using the Edison electric vehicle to test the system’s performance rigorously. This will involve developing and testing sophisticated sensor fusion algorithms that leverage IMU data to identify and mitigate noise in the ECG and PPG signals. Future work must also include expanding the participant cohort to validate the system’s efficacy across a broader demographic, as well as conducting longer-duration drives (>30 min) under varied road conditions (e.g., urban traffic, highways, and rough surfaces) to elicit more naturalistic driver behavior. Additionally, we are currently developing a technical concept for capacitive ECG sensing integrated into the vehicle seat. This technology is intended to serve as a redundant backup system to detect cardiac activity when steering wheel ECG signals are compromised by motion artifacts or temporary loss of hand contact. Comparative measurements between the dry-contact steering wheel and capacitive seat sensors will be the subject of a future study. The validated data acquisition platform will serve as the foundation for developing and training machine learning (ML) models that can accurately detect driver drowsiness and other adverse health states in real-time by analyzing physiological and steering behavior patterns. A critical component of this future development is the management of signal interruptions when the driver removes their hands from the wheel. While the current system utilizes hardware lead-off detection to flag and exclude invalid data, future iterations will use this status change to trigger a seamless transition to the redundant system (capacitive ECG sensing or camera-based monitoring) [58,59,60,61,62,63,64]. This multi-modal handover strategy ensures that the driver’s health profile remains continuous even during steering maneuvers that necessitate a change in grip. Because the prototype operates independently of the vehicle’s native electronics, its integration into research platforms, such as the Edison vehicle, is straightforward, allowing long-term driving studies to collect real-world data. This is crucial for developing ML algorithms for fatigue detection and prediction. Furthermore, this opens up possibilities for the unobtrusive, long-term health monitoring of professional drivers, a high-risk group for whom cardiovascular events are a leading cause of sudden death.

From a practical standpoint, this study contributes to the development of next-generation vehicle safety systems that can significantly reduce accidents caused by driver impairment or drowsiness and provide early warnings for acute medical events, thereby transforming the vehicle into a proactive guardian of driver well-being.

## 5. Conclusions

This paper presents the design, fabrication, and validation of an innovative smart steering wheel prototype for the unobtrusive, multimodal monitoring of a driver’s physiological state. We successfully demonstrated that the integration of dry-contact ECG, PPG, and IMU sensors into a single, ergonomic device is a viable approach for in-vehicle data acquisition. Our laboratory evaluations confirmed that the prototype successfully captured ECG and PPG signals of sufficient quality for reliable heart rate and HRV analyses, showing a strong correlation with a medical-grade reference device. Furthermore, initial on-road testing confirmed the system’s operational viability in a dynamic environment, highlighting both the challenges of motion artifacts and the fundamental robustness of the ECG sensor within the tested conditions. Future work will focus on extended real-world validation in the Edison research vehicle involving a diverse participant cohort to address inter-subject variability and ensure the system’s robustness across different physiological signal waveforms, including those associated with health conditions. This will be pursued alongside the development of advanced sensor fusion and machine learning algorithms. These will be designed to dynamically assess the quality of multimodal signals in real time and adaptively weight their contributions to improve the reliability of the final output.

## Figures and Tables

**Table 1:** Configuration parameters of the ECG sensor module (ADS1192).

Parameter	Value
Communication Interface	SPI
SPI Clock Speed	1 MHz
Sampling Rate	250 samples per second (SPS)
Bit resolution	16-bit (2-byte transfer)
Supply Voltage	3.3 V
Total gain range	21 to 252 (programmable)
Lead-off detection	Yes, the DC method on channel 2
Interrupt Pin	Enabled (active when data is ready)
High-pass filter cutoff	0.34 Hz (preprocessing stage)

**Table 2:** Configuration parameters of the PPG sensor module (MAX30102).

Parameter	Value
Communication Interface	I2C
I2C Clock Speed	400 kHz
Supply Voltage	3.3 V
LED Drive Current	~20 mA
Raw Sampling Rate	400 SPS
Averaging	4 samples
Effective Output Rate	100 SPS
Bit Resolution	16-bit (2-byte transfer)
Interrupt Pin	Enabled (active when data is ready)

**Table 3:** Configuration parameters of the IMU module (MPU6050).

Parameter	Value
Communication Interface	I2C
I2C Clock Speed	400 kHz
Supply Voltage	3.3 V
Measured Axes	Gyroscope *Z*-axis only
Full-Scale Range (Gyro)	±250 °/s
Sampling Rate	100 SPS
Bit Resolution	16-bit (2-byte transfer)
Interrupt Pin	Enabled (active when data is ready)

**Table 4:** Configuration parameters of the Bluetooth module (BM70).

Parameter	Value
Bluetooth Version	Bluetooth 4.2 (BLE)
Communication Interface	UART
UART Baudrate	921 600 Baud
Supply Voltage	3.3 V
Integrated Antenna	Chip antenna
Typical Range	~50 m (open-air conditions)
Max. Data Rate (BLE 4.2)	~1 Mbps (physical layer)

**Table 5:** Description of steering wheel data-frame structure.

Data Type	1st Byte (Type)	2nd Byte (Status)	3rd Byte (Data LSB)	4th Byte (Data MSB)
ECG	0x01	0x00 = Two hands contact; 0x01 or 0x02 = One/no hand contact	ECG sample (LSB)	ECG sample (MSB)
PPG	0x02	0x00	PPG sample (LSB)	PPG sample (MSB)
Gyroscope Z	0x03	0x00	Gyro Z sample (LSB)	Gyro Z sample (MSB)
Battery Level	0x05	0x00	Battery level sample (LSB)	Battery level sample (MSB)

**Table 6:** Performance metrics of peak detection in ECG signals within a 1 min interval.

Parameter	
Total peaks detected in both signals	81
True Positive peaks	81
False Positive peaks	0
Mean error of matched peaks	16.30 ms
Standard deviation of error	10.13 ms
Mean interbeat interval (Reference signal)	746.63 ms
Mean interbeat interval (Steering Wheel signal)	745.80 ms
Standard deviation of interbeat intervals (Reference signal)	24.05 ms
Standard deviation of interbeat intervals (Steering Wheel signal)	24.01 ms

**Table 7:** Performance metrics of peak detection in PPG signals within a 1 min interval.

Parameter	
Total peaks detected in both signals	80
True Positive peaks	80
False Positive peaks	0
Mean error of matched peaks	8.54 ms
Standard deviation of error	5.24 ms
Mean interbeat interval (Reference signal)	746.29 ms
Mean interbeat interval (Steering Wheel signal)	745.99 ms
Standard deviation of interbeat intervals (Reference signal)	25.22 ms
Standard deviation of interbeat intervals (Steering Wheel signal)	25.77 ms

## Data Availability

The data presented in this study are available on request from the corresponding author. The data are not publicly available due to privacy restrictions.
